# Considering the Bottom Edge Cutting Effect of the Carbon Fiber Reinforced Polymer Milling Force Prediction Model and Optimization of Machining Parameters

**DOI:** 10.3390/ma17235844

**Published:** 2024-11-28

**Authors:** Yiwei Zhang, Mengke Yan, Yushu Lai, Guixing Wang, Yifan Yang

**Affiliations:** 1 Chongqing Engineering Research Center for Advanced Intelligent Manufacturing Technology, Chongqing Three Gorges University, Chongqing 404000, China; yanmk2023@163.com (M.Y.); 18996295784@163.com (Y.Y.); 2 Chongqing Engineering Technology Research Center for Light Alloy and Processing, Chongqing Three Gorges University, Chongqing 404000, China; 3 School of Mechatronic Engineering, Southwest Petroleum University, Chengdu 610500, China; 15178940918@163.com

**Keywords:** CFRP, cutting force modeling, cutting force coefficients, genetic algorithms, parameter optimization

## Abstract

The milling force plays a pivotal role in CFRP milling. Modeling of the milling force is helpful to explore the changing law, optimize the processing parameters, and then reduce the appearance of defects. However, most of the existing models ignore the effect of the bottom edge. In this paper, the prediction of milling force in CFRP milling processes is taken as the research object. By analyzing the milling mechanism and considering the end milling cutter’s bottom cutting edge, the prediction model of milling force was established. Based on the experimental data and simulation data of milling force, the milling force coefficient was obtained by inverse calculation. Subsequently, the predicted cutting force was compared with the experimental cutting force, showing a maximum error of 14.5%, which is within a reasonable range, and the correctness of the model was verified. Furthermore, combined with the delamination damage and the milling force prediction model, a multi-objective optimization model of milling parameters was established, and the genetic algorithm was used to solve the model. The unidirectional carbon fiber plate with a fiber direction angle of 45° was selected as the optimization example. The minimum delamination damage was obtained under the cutting conditions of a spindle speed of 4903.1569 r/min, feed rate per tooth of 0.01 mm/z, and an axial depth of cut of 0.5 mm, and the experimental verification was carried out. The feasibility of the genetic algorithm in CFRP milling parameter optimization modeling was also verified.

## 1. Introduction

Carbon fiber reinforced polymers (CFRPs) are widely used in high-end aerospace equipment due to their advantages of specific strength, specific stiffness, corrosion resistance, light weight, etc., and are preferred materials for weight reduction and increased efficiency [1,2,3,4]. In the machining process, the cutting force has an important effect on the surface’s machining quality and processing efficiency [5]. Because CFRP is an inhomogeneous anisotropic material, the trend of cutting force is completely different under different fiber direction angles, and the functional relationship between the cutting force and various process parameters is not clear. For different fiber directions, it is impossible to predict the cutting force of CFRP through a unified mathematical expression [6].

The research on the changing law of cutting force and the establishment of prediction model can effectively control the cutting force and achieve more efficient machining [7]. Therefore, regarding CFRP milling force prediction, researchers have launched much research. Wang et al. [8] established an analytical cutting force model in the ball helical milling process, considering the cutting characteristics in the axial feed, based on the characteristics of helical milling technology and the geometric shape of the ball end cutter and the classical mechanical cutting force model. Shang et al. [9] analyzed the tool motion of helical milling and the geometry of the chip, and then the cutting force model was established. After that, the calibration method of cutting force coefficients was built. In the end, a series of cutting experiments was conducted to validate the cutting force model and the calibration method. Zhou et al. [10] built a 3D FEM model of CFRP helical milling to analyze the changing law of cutting force. Li et al. [11] established a dynamic cutting force model for helical milling that considered the cutting mechanism, and the cutting force of the periphery and bottom cutting edges was established. Chen et al. [12] established macroscopic and microscopic CFRP cutting models and found that the fiber direction was the main factor affecting the processing quality. The surface quality was poor when the fiber direction angle was 0° and 135°, while the surface quality under the directions of 90° and 45° was better. Ning et al. [13] established an analytical model of CFRP cutting force based on CFRP’s representative volume unit (RVE), considering the radius of the edge circle, and studied the mechanism of CFRP material removal in the cutting process. The theoretical model was proved by experiments. This proves that the analytical model built for the CFRP cutting force can provide values within a limited uncertainty, which is useful for many evaluations and machining applications. Wang et al. [14] developed, for the first time, a mechanistic feeding-directional cutting force model for RUM end surface grinding of CFRP composites with elliptical ultrasonic vibration. The presented model was validated through comparisons between the predicted cutting forces and experimental results under each input–variable combination. Kim et al. [15] used a CFRP cutting force model to analyze the cutting force of upper and lower milling. Chip formations were predicted via simulations of the fiber cutting angle. Zhang et al. [16] established a novel predictive cutting force model of helical milling for unidirectional CFRP. Based on the cutting parameters, the dynamic cutting angle for the fiber and the corresponding cutting states of the fiber during the cutting process were analyzed, and the number and length of cutting edges involved in cutting were calculated. Sheikh-Ahmad et al. [17] utilized the mechanistic modeling approach in combination with neural network data fitting for simulating the cutting forces in the milling of unidirectional carbon fiber reinforced polymers (UD-CFRP). A method was proposed for predicting the cutting forces for tools with a complex geometry by transforming the specific cutting energies from orthogonal cutting to oblique cutting and accounting for the effects of the rake angle and edge radius. Ning et al. [18] established a back propagation (BP) neural network model of cutting force and edge force coefficients. The model considers the effects of the instantaneous uncut chip thickness, fiber cutting angle, spindle speed, and axial depth of the cut. In summary, although there are many studies on modeling cutting force at home and abroad, most scholars only focus on the contribution of the tool’s side edge to cutting force but ignore the influence of the tool’s bottom edge on cutting force. In fact, the tool’s bottom edge is always involved in cutting. Wan et al. [19] studied the size effect of the tool’s bottom edge when cutting a titanium alloy and proved that the contribution of the tool’s bottom edge to the cutting force cannot be ignored.

About machining parameter optimization, because conflicting processing solutions are given for each processing parameter, it is necessary to obtain a multi-objective optimization solution. Ozsoy et al. [20] optimized cutting parameters such as the cutting speed, feed rate, and cooling type. Regression equations were obtained with the response surface method (RSM). Amani et al. [21] modeled the drilling process using the response surface methodology (RMS) and artificial neural networks (ANNs). RMS and ANN results were compared. ANNs were closer to the experiment than the RMS. Barik et al. [22] employed multi-criteria decision-making techniques, such as MOORA, TOPSIS, and VIKOR, to identify the optimal combination of parameters for minimizing defects and enhancing drilling efficiency. Currently, the genetic algorithm (GA) has rarely been used to optimize cutting parameters in CFRP processing. Moreover, the Pareto optimal solution is often referred to as the Pareto frontier (Pareto front), which is more than the optimal solution set [23]. Above all, this study established a cutting force prediction model considering the cutting effect of the end milling cutter’s bottom edge and used experimental and simulation data to solve the cutting force coefficient. By analyzing the influence of the fiber direction angle, spindle speed, feed rate per tooth, axial depth of cutting force on cutting force, the relationship between the cutting force coefficient and these processing parameters was obtained. The accuracy of the cutting force prediction model was verified by experiment. Building upon this foundation, the optimal solution was achieved through the application of a genetic algorithm, demonstrating its feasibility in optimizing CFRP milling parameters. Consequently, this study holds significant implications for more accurate predictions of milling force and the optimization of milling parameters under specific multi-objective conditions.

## 2. Modeling Method and Experiment

### 2.1. Establish a Milling Force Prediction Model

During CFRP milling, variations in the instantaneous cutting thickness at the same edge due to the tool’s helix angle causes fluctuating cutting forces [24]. The cutting force in the milling process is affected by the side edge and the bottom edge of the tool [25]. The current literature on CFRP milling force mainly focuses on the side edge of the tool, ignoring the contribution of the bottom edge to the cutting force. Therefore, in this paper, the instantaneous rigid force model is used to model the side-edge and bottom-edge cutting force.

#### 2.1.1. Side-Edge Cutting Force Model

**Figure 1:**
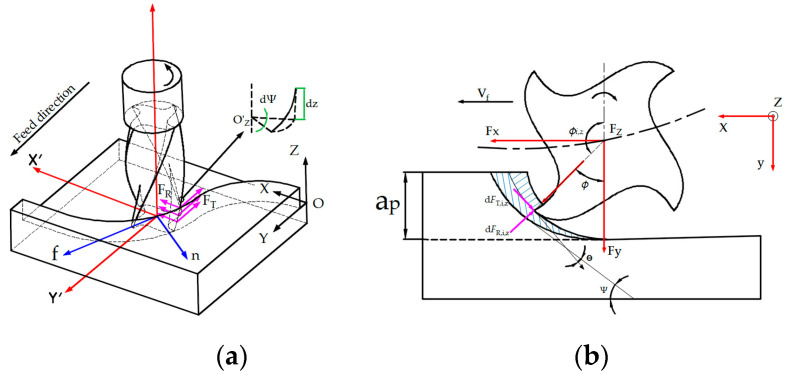
Model for the end milling of a curved surface. (**a**) Geometries and mechanics of the cutting process. (**b**) Cutting forces related to the infinitesimal cutter element.

The cutting edge of the milling cutter is segmented into a finite number of equal-height micro-cutting edges along its axial direction. The force on the cutting element is equal to the product of the cutting force coefficient and the area of the element [26]. The total cutting force of each micro-cutting edge can be divided into its tangential, radial and axial components (Figure 1), which are represented by $F_{t}$, $F_{r}$, and $F_{a}$, respectively. The expression is shown in Equation (1)

(1) $$\left\{\begin{array}{c} dF_{tc,i}(\phi_{i}(z))=[K_{tc}h_{i}(\phi_{i}(z))+K_{te}]dz\\ dF_{rc,i}(\phi_{i}(z))=[K_{rc}h_{i}(\phi_{i}(z))+K_{re}]dz\\ dF_{ac,i}(\phi_{i}(z))=[K_{ac}h_{i}(\phi_{i}(z))+K_{ae}]dz \end{array}\right.$$

where *i* denotes the cutting edge number *i*; $\phi_{i}$ denotes the instantaneous milling angle; hi denotes the *i*-th tooth of the transient chip thickness, where $h_{i}(\phi_{i}(z))=f_{z}\sin\phi_{i}(z)$; $K_{tc}$, $K_{rc}$, and $K_{ac}$ denote the tangential, radial, and axial cutting force coefficients, respectively; and $K_{te}$, $K_{re},\;and\;K_{ae}$ denote the cutting edge coefficients of the tangential, radial, and axial cutting forces, respectively.

However, due to the helical angle of the helical end mill, the instantaneous milling depth at any point on a single tooth is different, and the instantaneous cut-in angle is also different. The rotation angle at any point lags behind the rotation angle of the bottom cutting edge. Assuming that the instantaneous cut-in angle of the bottom edge is ϕ, the instantaneous cut-in angle of the i-th bottom cutting edge can be obtained by Equation (2):

(2) $$\phi_{i}=\phi +i\phi_{p};i=0,1,2,\ldots N$$

Then the tool rotation angle at the axial cutting depth *z* of the *i*-th tooth is as follows:

(3) $$\phi_{i}\left(z\right)=\phi +i\phi_{P}-k_{\beta }z$$

Within Equation (3), $\phi_{p}=\frac{2\pi }{N}$; ${\;k}_{\beta }z\;$ denotes the lag angle at *z* for a single cutter. The corresponding lag angle ξ at the axis depth *z* can be expressed as follows: $\xi =\frac{2z\tan\beta }{D}.$ So $k_{\beta }=(2\tan\beta )/D$, *β* is the cutter’s helix angle, and *D* is the diameter of the cutter.

#### 2.1.2. Bottom-Edge Cutting Force

According to Wan et al., the material removal process occurring at the bottom edge closely resembles that of the side edge during the milling operation [19], which is also expressed as the cutting force in the radial, tangential, and axial directions. The expression is as follows (Equation (4))

(4) $$\left\{\begin{array}{c} dF_{tb}=K_{tb}h_{b}dz\\ dF_{rb}=K_{rb}h_{b}dz\\ dF_{ab}=K_{ab}h_{b}dz \end{array}\right.$$

where $K_{tb}$, $K_{rb}$, and $K_{ab}$ are the cutting force coefficients of the tangential, radial, and axial bottom edges; $h_{b}$ represents the instantaneous chip thickness of the bottom edge. The expression $h_{b}$ is $h_{b}=f_{z}\sin\phi$.

Then the total cutting force can be expressed as:

(5) $$\left\{\begin{array}{c} dF_{t}=dF_{tc}+dF_{tb}\\ dF_{r}=dF_{rc}+dF_{rb}\\ dF_{a}=dF_{ac}+dF_{ab} \end{array}\right.$$

The experimental data are the cutting forces in the *x*, *y*, and *z* directions; therefore, in order to facilitate the calculation, the cutting force should be transformed into the rectangular coordinate system [7]:

(6) $$\left\{\begin{array}{c} dF_{x,i}(\phi_{i}(z))=-dF_{t,i}\cos(\phi_{i}(z))-dF_{r,i}\sin(\phi_{i}(z))\\ dF_{y,i}(\phi_{i}(z))=+dF_{t,i}\cos(\phi_{i}(z))-dF_{r,i}\sin(\phi_{i}(z))\\ dF_{z,i}(\phi_{i}(z))=+dF_{a,i} \end{array}\right.$$

The micro-element cutting forces are integrated along the axial direction, and the milling force on the *i*-th cutting edge can be expressed as:

(7) $$F_{q,i}(\phi_{i}(z))=\int_{z_{i,1}(\phi_{i}(z))}^{z_{i,2}(\phi_{i}(z))}dF_{q}(\phi_{i}(z))dz,q=x,y,z$$

Substituting Equation (6) into Equation (7) results in:

(8) $$F_{x,i}(\phi_{i}(z))={\left\{\begin{array}{c} \frac{f}{4k_{\beta }}[-k_{tc}\cos2\phi_{i}(z)-k_{tb}\cos2\phi +k_{rc}[2\phi_{i}(z)-\sin2\phi_{i}(z)]\\ +k_{rb}[2\phi_{i}(z)-\sin2\phi_{i}(z)]]+\frac{1}{k_{\beta }}[k_{te}\sin\phi_{i}(z)-k_{re}\cos\phi_{i}(z)] \end{array}\right\}}_{z_{i,1}(\phi_{i}(z))}^{z_{i,2}(\phi_{i}(z))}$$

(9) $$F_{y,i}(\phi_{i}(z))={\left\{\begin{array}{c} -\frac{f}{4k_{\beta }}[k_{rc}\cos2\phi_{i}(z)+k_{rb}\cos2\phi +k_{tc}[2\phi_{i}(z)-\sin2\phi_{i}(z)]\\ +k_{tb}[2\phi_{i}(z)-\sin2\phi_{i}(z)]]+\frac{1}{k_{\beta }}[k_{te}\cos\phi_{i}(z)+k_{re}\sin\phi_{i}(z)] \end{array}\right\}}_{z_{i,1}(\phi_{i}(z))}^{z_{i,2}(\phi_{i}(z))}$$

(10) $$F_{z,i}(\phi_{i}(z))=\frac{1}{k_{\beta }}[k_{ac}f\cos\phi_{i}(z)+k_{ab}f\cos\phi_{i}(z)-k_{ae}\phi_{i}(z){]}_{z_{i,2}(\phi_{i}(z))}^{z_{i,2}(\phi_{i}(z))}$$

Then, when the instantaneous milling angle is ϕ, the milling force of *N* teeth in one cycle is:

(11) $$F_{x}\left(\phi \right)=\sum_{i=1}^{N}F_{xi};F_{y}\left(\phi \right)=\sum_{i=1}^{N}F_{yi};F_{z}\left(\phi \right)=\sum_{i=1}^{N}F_{zi}$$

In the case of certain machining parameters, the volume of material removed by a single tooth of the tool in a cycle is fixed and not affected by the helix angle, so the helix angle has no effect on the average cutting force [27]. The average force in a cycle is calculated as follows.

(12) $$\bar{F_{q}}=\frac{1}{\phi_{a}}\int_{\phi_{st}}^{\phi_{ex}}F_{q}(\phi )d\phi$$

The average milling force of one cycle after integration is:

(13) $$\bar{F_{x}}={\left\{\begin{array}{c} \frac{Na_{p}f_{z}}{8\pi }[k_{tc}\cos2\phi -k_{rc}[2\phi -\sin2\phi ]]+\frac{Na_{b}f_{z}}{8\pi }[k_{tb}\cos2\phi -k_{rb}[2\phi -\sin2\phi ]]\\ +\frac{Na_{p}}{2\pi }[-k_{te}\sin\phi +k_{re}\cos\phi ] \end{array}\right\}}_{\phi_{st}}^{\phi_{ex}}$$

(14) $$\bar{F_{y}}={\left\{\begin{array}{c} \frac{Na_{p}f_{z}}{8\pi }[k_{rc}\cos2\phi +k_{tc}[2\phi -\sin2\phi ]]+\frac{Na_{b}f_{z}}{8\pi }[k_{rb}\cos2\phi +k_{tb}[2\phi -\sin2\phi ]]\\ -\frac{Na_{p}}{2\pi }[k_{te}\cos\phi +k_{re}\sin\phi ] \end{array}\right\}}_{\phi_{st}}^{\phi_{ex}}$$

(15) $$\bar{F_{z}}={\left\{-\frac{Na_{p}}{2\pi }k_{ac}f_{z}\cos\phi -\frac{Na_{b}}{2\pi }k_{ab}f_{z}\cos\phi +\frac{Na_{p}}{2\pi }k_{ae}\phi \right\}}_{\phi_{st}}^{\phi_{ex}}$$

### 2.2. Experiment

#### 2.2.1. Finite Element Simulation Experiments

In the finite element simulation analysis, the material model is an important factor to ensure the correctness of the finite element simulation results for CFRP milling. In order to accurately simulate the damage of CFRP milling, it is necessary to clarify the characteristics of CFRP materials and the initial failure criteria. Carbon fiber composites are regarded as anisotropic linear elastic materials [28]. The elastic constitutive model of the materials is as follows

(16) $$\left[\begin{array}{c} \sigma_{11}\\ \sigma_{22}\\ \sigma_{33}\\ \tau_{12}\\ \tau_{23}\\ \tau_{13} \end{array}\right]=\left[\begin{array}{cccccc} C_{11}^{0} & C_{12}^{0} & C_{13}^{0} & 0 & 0 & 0\\ C_{12}^{0} & C_{22}^{0} & C_{23}^{0} & 0 & 0 & 0\\ C_{13}^{0} & C_{23}^{0} & C_{33}^{0} & 0 & 0 & 0\\ 0 & 0 & 0 & C_{44}^{0} & 0 & 0\\ 0 & 0 & 0 & 0 & C_{55}^{0} & 0\\ 0 & 0 & 0 & 0 & 0 & C_{66}^{0} \end{array}\right]\left[\begin{array}{c} \varepsilon_{11}\\ \varepsilon_{22}\\ \varepsilon_{33}\\ \gamma_{12}\\ \gamma_{23}\\ \gamma_{13} \end{array}\right]$$

where

(17) $$\begin{array}{c} C_{11}^{0}=E_{11}(1-\upsilon_{23}\upsilon_{32})\Gamma ,\;C_{22}^{0}=E_{22}(1-\upsilon_{13}\upsilon_{31})\Gamma ,\\ C_{33}^{0}=E_{33}(1-\upsilon_{12}\upsilon_{21})\Gamma ,\;C_{12}^{0}=E_{11}(\upsilon_{21}+\upsilon_{31}\upsilon_{23})\Gamma ,\\ C_{23}^{0}=E_{22}(\upsilon_{32}+\upsilon_{12}\upsilon_{31})\Gamma ,\;C_{13}^{0}=E_{33}(\upsilon_{31}+\upsilon_{21}\upsilon_{32})\Gamma ,\\ C_{44}^{0}=G_{12},\;C_{55}^{0}=G_{12},\;C_{66}^{0}=G_{13},\\ \Gamma =1/(1-\upsilon_{12}\upsilon_{21}-\upsilon_{23}\upsilon_{32}-\upsilon_{13}\upsilon_{31}-2\upsilon_{21}\upsilon_{32}\upsilon_{13}) \end{array}$$

where $C_{ij}^{0}$ denotes the stiffness coefficient, $E_{ij}$ denotes the elastic modulus (Gpa), and $\upsilon_{ij}$ denotes the Poisson ratio.

The Hashin failure criteria include the tensile and compressive failure modes of the fiber and the matrix. However, the relevant literature shows that the Hashin failure criteria cannot accurately analyze the matrix damage. Therefore, the Hashin criterion is used to judge the failure of fibers, and the Puck criterion is used to judge the failure of the matrix [29]. In order to simulate the CFRP milling process more realistically, cohesive elements were connected between the fiber layers with a small thickness. The constitutive model of the cohesive element material, the failure criterion, and the form of damage evolution are described in detail in reference [30]. The mechanical properties parameters of CFRP unidirectional plate material are presented in Table 1.

The CFRP milling process involves stress and strain in three-dimensional space. The Hashin failure criterion of ABAQUS 2021 is only applicable to the shell element in two-dimensional space, ignoring the change in stress in the thickness direction, which cannot meet the requirements of three-dimensional macro-milling simulation of CFRP. Therefore, the secondary development of finite element simulation is needed. In this paper, a VUMAT subroutine is written, based on Fortran language. The VUMAT subroutine includes three parts: the elastic phase of the CFRP material, the damage initiation failure criteria, and the material’s phase of damage evolution. The subroutine’s flow is as follows. Firstly, ABAQUS automatically calculates the steady-state incremental step, reads the material-related parameters entered by the user’s material window in the property module, and stores them in the props array to read the strain increment. The material properties are introduced into the material’s constitutive model, the elastic stiffness matrix is calculated, and the stress–strain and state variables are updated. According to Hashin’s and Puck’s initial failure criteria, the subroutine determines whether the stress meets the failure conditions. If the initial failure criterion is met, the stiffness degradation matrix is calculated and the stress is recalculated. If the unit fails completely, the unit is deleted. If the failure condition is not met, the subroutine is returned to recalculate the stress strain.

**Figure 2:**
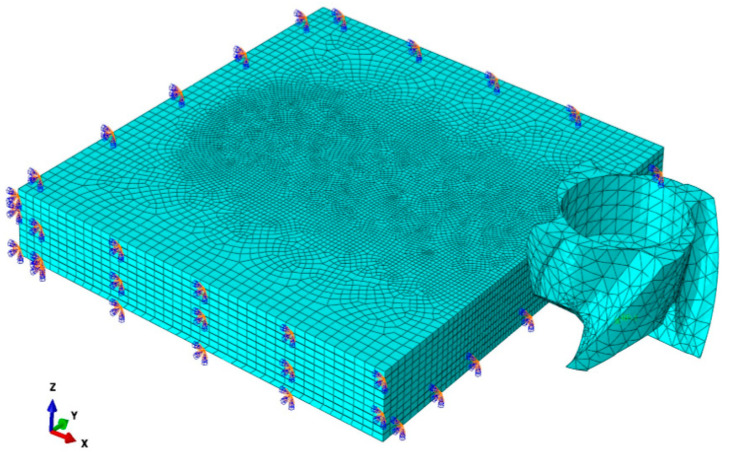
Drawing of the model’s assembly.

The finite element model for three-dimensional CFRP milling included the cutter and the workpiece. Due to the simple structure of the workpiece, it was directly modeled in ABAQUS software. The model’s dimensions were 15 mm × 16 mm × 2.09 mm, comprising 10 layers, each with a thickness of 0.2 mm, connected by thin cohesive elements between the layers, and the base of the workpiece was firmly fixed. A geometric model of the tool was drawn using Solidworks and imported into ABAQUS software. The tool consisted of a handle and teeth. If the tool model was excessively large, it increased the computational volume and efficiency significantly, so only the 5 cm length of the tooth portion was retained. To facilitate subsequent load application and output variables, a reference point was set at the center of the tool and bound to the tool. The assembly diagram of the model is shown in Figure 2.

The model was simplified by assuming the cutting tool to be a rigid body; CFRP was assumed to be homogeneous, with the fibers and resin adhering tightly, and contact resistance was ignored. Carbon fibers were evenly distributed within the resin, with no impurities, delamination, or other defects during preparation, and the layers were assumed to be strongly bonded to each other, with contact resistance between layers also being ignored. During CFRP cutting, the unit deformation is significant, and the grid density affects the calculation efficiency and accuracy of simulation model. In order to accurately predict the change in stress and the machining damage during CFRP milling and improve the calculation efficiency, away from the cutting area part, the grid size was 0.4 mm, and the cutting area’s grid size was 0.1 mm; the element type was hexahedral. The structural formula was used to divide the element. The grid element type was eight-node hexahedron linear reduction integral elements, C3D8R. The cohesive element type was also hexahedral, and the grid was divided by the sweeping method. The grid attribute is eight-node cohesive force element (COH3D8). The total number of grids for the CFRP workpiece was 148,257. The cutter grid was divided into 0.5 mm, with a tetrahedral unit type, utilizing a free-form division method. The cutter grid was a four-node linear entity (C3D4), with a total of 4546 grids.

The contact between the workpiece and cutter was set to point-surface contact, with the cutter being the primary surface and the workpiece the secondary surface. The normal contact was rigid, whereas the tangential contact was frictional, defined with a penalty functional, expressed as Equation (18)

(18) $$\tau_{n}=\mu \times \sigma_{n}$$

where $\tau_{n}$ denotes normal stress (MPa), μ denotes the friction factor, and $\sigma_{n}$ denotes tangential stress.

The friction coefficient is affected by the fiber direction angle. Under different fiber direction angles (0°, 45°, 90°, 135°), the friction factor of the workpiece and the tool was 0.2, 0.4, 0.6, and 0.2, respectively. The simulation’s test parameters are as shown in Table 2.

#### 2.2.2. Milling Experiment

**Figure 3:**
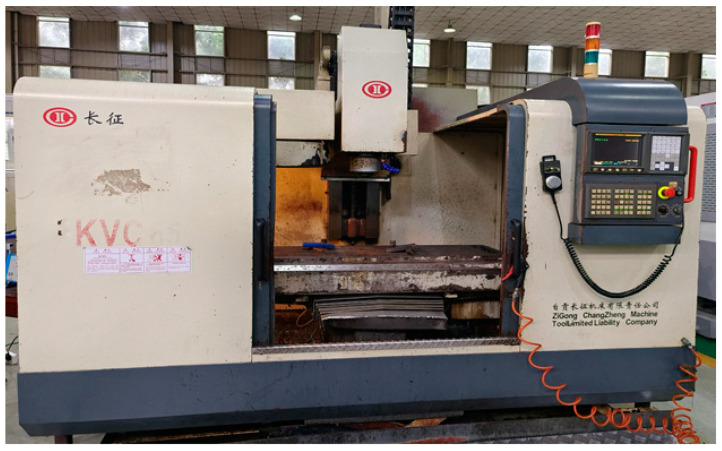
KVC650 CNC milling machine.

The experiment utilized the Changzheng KVC650 CNC milling machine (Zhongshan China) for the milling of CFRP, as shown in Figure 3. The maximum speed of the machine was 8000 rpm, and the maximum feed speed was 8000 mm/min. Due to the potential impact of the cutting fluid on the properties of the carbon fiber composite material, dry cutting was employed in processing the unidirectional CFRP plates. The T800 unidirectional carbon fiber composite plate with a thickness of 4 mm was selected as the experimental workpiece. This paper selected a 4-edge helix end milling cutter and the helix angle of the tool was 45°, the diameter was 6 mm, and the cutter was made of cemented carbide. In the experiment, the model Kistler9257B dynamometer (Winterthur, Switzerland) was used to measure the milling force, and the model of the charge amplifier was Kistler5070A12100.

**Figure 4:**
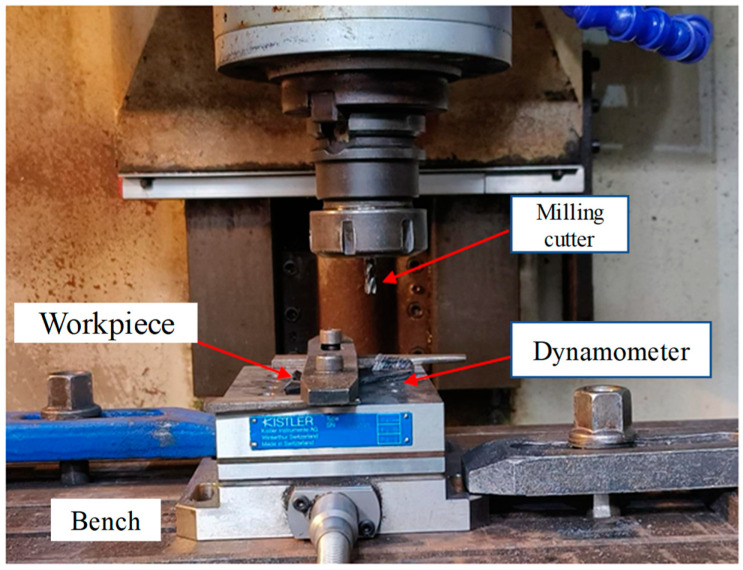
Experimental platform.

Figure 4 shows the experimental platform. The workpiece was fixed on the processing platform with screws.

The machining parameters of the milling experiment were consistent with those of simulation experiment.

#### 2.2.3. Verification of the CFRP Milling Simulation Model

**Figure 5:**
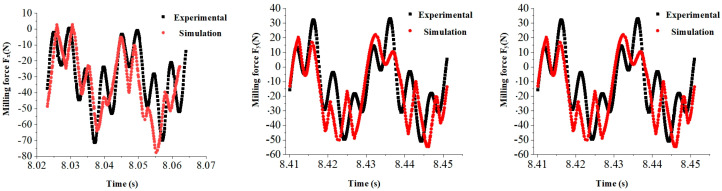
Experimental and simulation values of instantaneous cutting force at a fiber direction angle of 45°.

**Figure 6:**
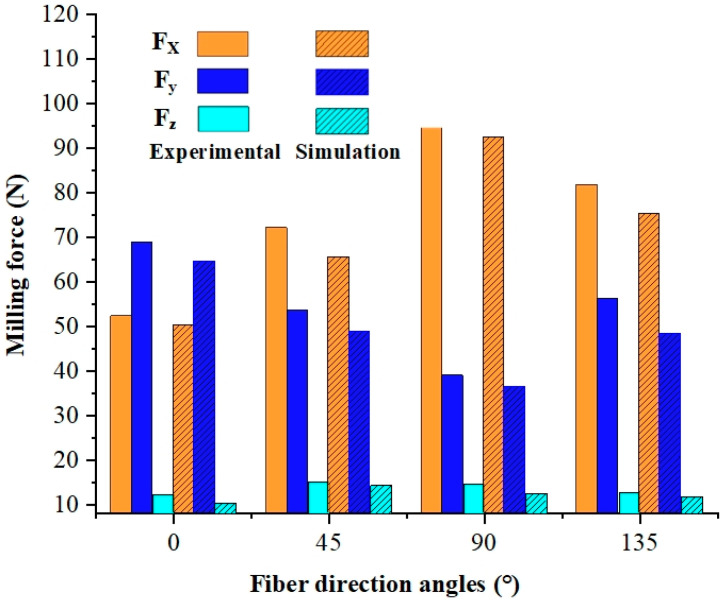
Average value of milling force under different fiber direction angles.

Data analysis enables us to obtain both the experimental and simulated values for cutting force. Figure 5 displays the instantaneous cutting force values at a fiber direction angle of 45°, indicating good agreement between the experimental and simulation results regarding their change patterns over time. Average milling force calculations were performed within stable cutting regions during milling (as shown in Figure 6), resulting in a maximum relative error of only 15.04% when comparing the experimental values with the predicted ones from simulations.

From the results above, we could know the correctness of this simulation model, so we could use the combination of simulation and experiments to obtain data.

#### 2.2.4. Experimental Setup

Using the experimental conditions above for the milling experiments, and referring to the existing literature, we determined the commonly used range of spiral milling processing parameters. According to the commonly used range of spiral milling processing parameters for milling the full experiment, we obtained more accurate bottom-edge axial cutting force coefficients under different cutting direction angles, which were used to predict the axial force in the subsequent spiral milling process. The experimental parameters of CFRP milling are shown in Table 3, and the experimental parameters of simulated milling are shown in Table 4.

## 3. Results and Discussion

### 3.1. Calculation of the Cutting Force Coefficient

In the milling force model, the milling force coefficient is a pivotal parameter, its accurate determination being imperative. The accuracy of the milling force coefficient influences the precision of the milling force prediction model. Assuming that the milling force in each direction is known, the milling force coefficient is solved by the inverse method. In the process of blind groove milling, the cutter’s milling entrance angle is $\phi_{st}$ = 0°, and the milling exit angle is $\phi_{ex}$ = 180°. Substitution in Equations (13)–(15) is used to obtain Equation (19):

(19) $$\left\{\begin{array}{c} \bar{F_{x}}=-\frac{Na_{p1}}{4}K_{rc}f_{z}-\frac{Na_{pb}}{4}K_{rb}f_{z}-\frac{Na_{p1}}{\pi }K_{re}\\ \bar{F_{y}}=+\frac{Na_{p1}}{4}K_{tc}f_{z}+\frac{Na_{pb}}{4}K_{tb}f_{z}+\frac{Na_{p1}}{\pi }K_{te}\\ \bar{F_{z}}=\frac{Na_{p1}}{\pi }K_{ac}f_{z}+\frac{Na_{pb}}{\pi }K_{ab}f_{z}+\frac{Na_{p1}}{2}K_{ae} \end{array}\right.$$

According to Equation (19), when the axial cutting depth and the number of tool teeth are fixed, the average cutting force can be expressed as a functional relationship related to the feed rate:

(20) $$\bar{F_{q}}=\bar{F_{qm}}f_{z}+\bar{F_{qe}},q=x,y,z$$

(21) $$\left\{\begin{array}{c} \bar{F_{xm}}=-\frac{Na_{p1}}{4}K_{rc}-\frac{Na_{pb}}{4}K_{rb};\;\bar{{F_{x}}_{e}}=-\frac{Na_{p1}}{\pi }K_{re}\\ \bar{{F_{y}}_{m}}=+\frac{Na_{p1}}{4}K_{tc}+\frac{Na_{pb}}{4}K_{tb};\;\bar{{F_{y}}_{e}}=\frac{Na_{p1}}{\pi }K_{te}\\ \bar{{F_{z}}_{m}}=+\frac{Na_{p1}}{\pi }K_{ac}+\frac{Na_{pb}}{\pi }K_{ab};\;\bar{{F_{a}}_{e}}=\frac{Na_{p1}}{2}K_{ae} \end{array}\right.$$

Solving for $K_{rc}$, $K_{rb}$, $K_{rc}$, $K_{tb}$, $K_{ac}$, and $K_{ab}$ will be in different coaxial cutting depth conditions $\bar{F_{qm}}$, recorded as $\bar{{F_{qm}}_{1}}$ and $\bar{{F_{qm}}_{2}}$:

(22) $$\left\{\begin{array}{c} \bar{F_{xm1}}=-\frac{Na_{p1}}{4}K_{rc}-\frac{Na_{pb}}{4}K_{rb};\bar{{F_{x}}_{m2}}=-\frac{Na_{p2}}{4}K_{rc}-\frac{Na_{pb}}{4}K_{rb}\\ \bar{{F_{y}}_{m1}}=+\frac{Na_{p1}}{4}K_{tc}+\frac{Na_{pb}}{4}K_{tb};\bar{{F_{y}}_{m2}}=\frac{Na_{p2}}{4}K_{tc}+\frac{Na_{pb}}{4}K_{tb}\\ \bar{{F_{z}}_{m1}}=+\frac{Na_{p1}}{\pi }K_{ac}+\frac{Na_{pb}}{\pi }K_{ab};\bar{{F_{z}}_{m2}}=\frac{Na_{p2}}{\pi }K_{ac}+\frac{Na_{pb}}{\pi }K_{ab} \end{array}\right.$$

The cutting force coefficient is:

(23) $$\left\{\begin{array}{c} K_{rc}=\frac{4(\bar{{F_{x}}_{m2}}-\bar{{F_{x}}_{m1}})}{N(a_{p1}-a_{p2})};K_{rb}=\frac{4(\bar{{F_{x}}_{m2}}\;a_{p1}-\bar{{F_{x}}_{m1}\;}a_{p2})}{N(a_{p2}-a_{p1})a_{pb}};\;K_{re}=-\frac{\bar{{F_{x}}_{e}}\pi }{Na_{p1}}\\ K_{tc}=\frac{4(\bar{{F_{y}}_{m2}}-\bar{{F_{y}}_{m1}})}{N(a_{p2}-a_{p1})};K_{tb}=\frac{4(\bar{{F_{y}}_{m2}}\;a_{p1}-\bar{{F_{y}}_{m1}\;}a_{p2})}{N(a_{p2}-a_{p1})a_{pb}};\;\;K_{te}=\frac{\bar{{F_{y}}_{e}}\pi }{Na_{p1}}\\ K_{ac}=\frac{N(\bar{{F_{z}}_{m2}}-\bar{{F_{z}}_{m1}})}{\pi (a_{p2}-a_{p1})};K_{ab}=\frac{4(\bar{{F_{z}}_{m2}}\;a_{p1}-\bar{{F_{z}}_{m1}\;}a_{p2})}{N(a_{p2}-a_{p1})a_{pb}};\;K_{ae}=\frac{2\bar{{F_{a}}_{e}}}{Na_{p1}} \end{array}\right.$$

### 3.2. Analysis of the Experimental Results

**Figure 7:**
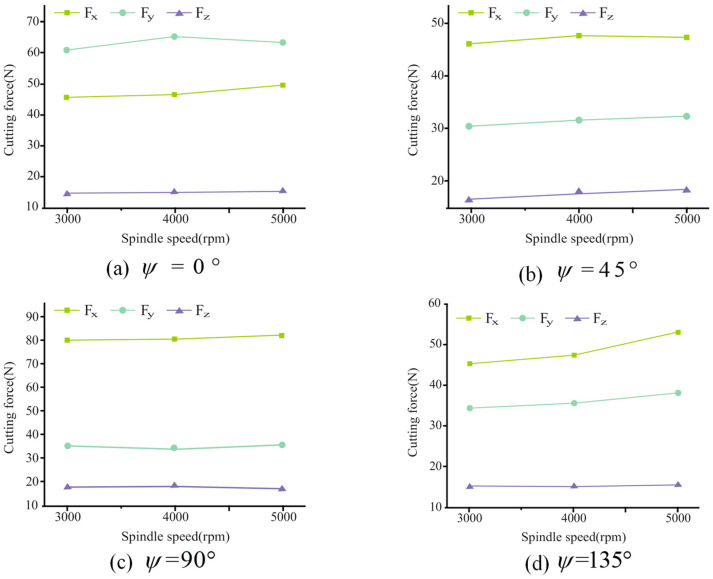
Influence of spindle speed on cutting force.

**Figure 8:**
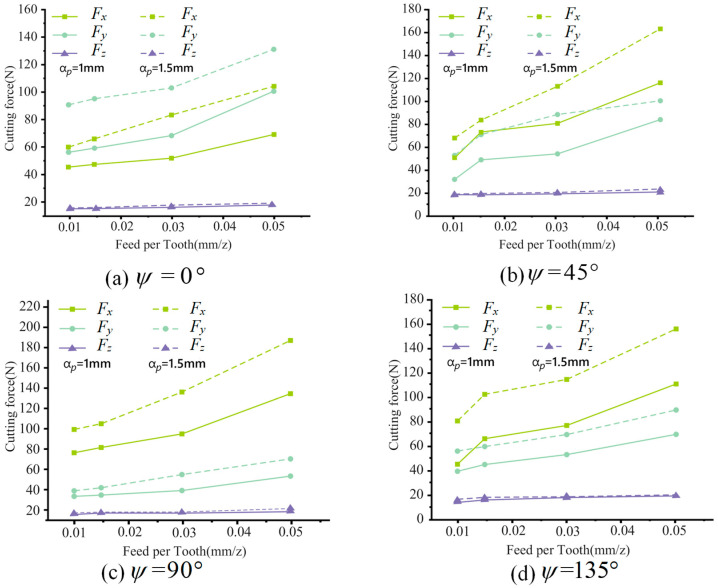
Effect of feed rate per tooth on cutting force.

The cutting force along the X, Y, and Z axes during the milling process can be measured by the dynamometer, and the cutting force in all three directions changes periodically. Under the cutting conditions of a 0.01 mm/z feed rate per tooth and a 1 mm axial cutting depth, the influence of spindle speed on the cutting force is shown in Figure 7. It can be seen from the figure that the cutting force increases slightly with an increase in the spindle speed, indicating that the cutting force is less affected by the spindle speed. Under the cutting conditions of a spindle speed of 4000 r/min and axial depths of 1 mm and 1.5 mm, the influence of the feed rate per tooth on cutting force is shown in Figure 8. It can be seen from the figure that the cutting force in the X, Y, and Z directions increase with the increase in the feed rate per tooth. The maximum increase in cutting force in the X and Y direction is 46 N, while the increase in cutting force in the Z direction is small.

According to Equation (20), the cutting force can be regarded as a linear relationship related to the feed rate per tooth. The cutting force data of Figure 8 were fitted, and the fitting function can be expressed as

(24) y=a+bx

where a denotes the intercept of the fitted line, and b denotes the slope of fitting line.

The fitting coefficients obtained under different fiber direction angles are shown in Table 5 and Table 6. Data from Table 5 and Table 6 substituted into Equation (22) obtained the coefficient of cutting force. The cutting force coefficients of the results are as shown in Table 7.

### 3.3. Verifying the Accuracy of the Milling Force Model

In order to prove the correctness of the cutting force prediction model, it was necessary to compare the experimental results of cutting force with the predicted results. The experiment parameters of milling are shown in Table 8.

The expression of relative error of milling force in the x, y, and z directions is as follows

(25) $$\varepsilon =\left|\frac{F_{e}-F_{m}}{F_{e}}\right|\times 100\%$$

where ε denotes the relative error, $F_{e}$ denotes the experimental value of milling force, and $\;F_{m}$ denotes the predicted value of milling force.

**Figure 9:**
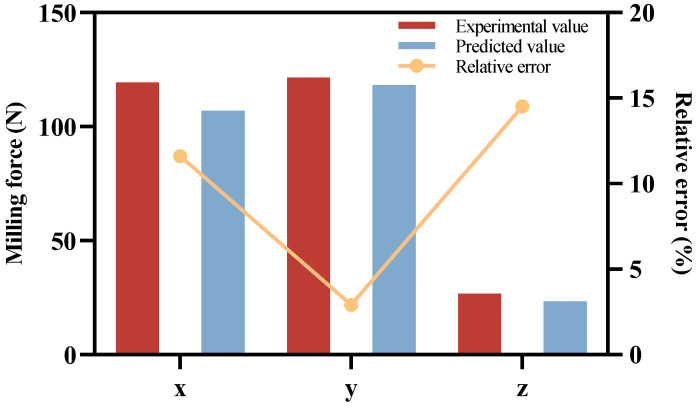
The relative error between the experimental and predicted milling force.

Table 9 and Figure 9 show the relative error between the experimental value and the predicted value of milling force, which proves that the established milling force prediction model can better predict the size of the average milling force. Its maximum value was 14.5% in the axial direction.

This is because the tangential force (F_tc_) and the radial force (F_rc_) will be generated when cutting takes place on the side edge of the tool. When the tool moves with a helix angle λ, the actual tangential force of the side edge is along the direction of the cutting speed of the tool. Therefore, the axial component of the tangential and radial force of the side edge will be generated. In addition, the method of filtering the measured force signal can lead to the loss of the partial signal signature and lead to error. The superposition method used to calculate the instantaneous milling force of multidirectional CFRP is an approximate equivalent method, which also leads to the existence of errors.

## 4. Parameter Optimization of the CFRP Milling Process

Machining parameters are important factors affecting the milling force and delamination damage of CFRP. The quality and efficiency of CFRP milling can be improved by selecting suitable machining parameters [31]. The responses obtained were the cutting force, delamination factor, and material removal rate. Because the machining responses are conflicting in nature, the problem was formulated as a multi-objective optimization problem. The unidirectional carbon fiber plate with fiber direction angle of 45° was selected as the example for optimization.

### 4.1. Establishment of the Multi-Objective Optimization Model

**Figure 10:**
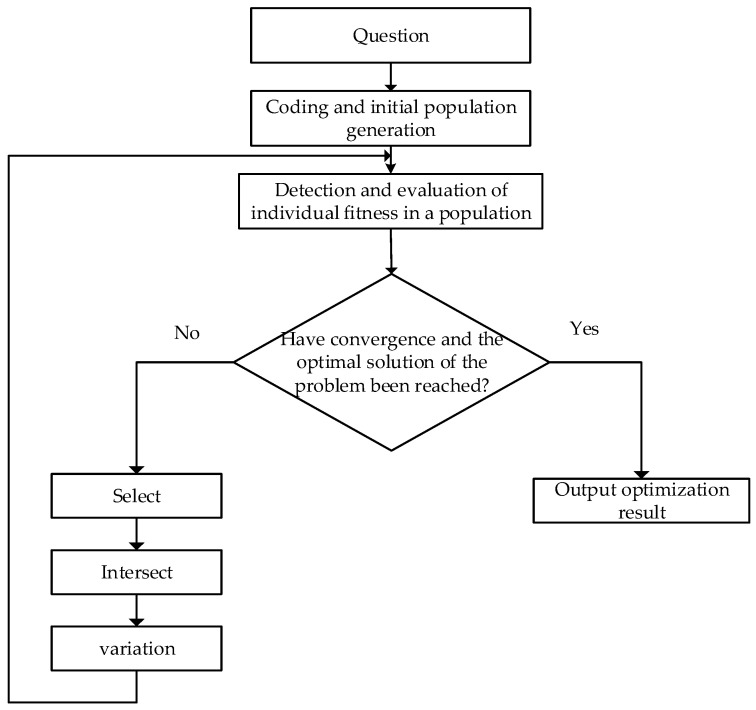
Calculation process of the genetic algorithm.

The genetic algorithm (GA) is a method used to search the global optimal solution by simulating the natural evolution process, which has the characteristics of strong robustness and high efficiency [32]. Compared with other algorithms, the genetic algorithm can obtain the ideal global optimal solution and has unique advantages in solving nonlinear and multi-objective function optimization problems, as it is simple in application and efficient in calculation. Figure 10 shows the calculation process of the genetic algorithm.

#### 4.1.1. Determination of Optimization Variables

The objective function is usually an expression composed of known or unknown parameters. The parameters that need to be determined in the optimization process are design variables. The principles to be followed when selecting design variables are as follows.

- There is a certain connection with the objective function, and it has a greater impact on the objective function.
- The parameters should be independent and of practical significance.
- First and second, reduce the number of variables as much as possible to simplify the optimization problem.

According to the research above, the spindle speed *n*, the feed rate per tooth $f_{z}$, and the axial cutting depth $a_{p}$ have an impact on CFRP machining damage and milling force, so they are used as optimization variables [33]. We denote each the three variables as:

(26) $$X=\left[x_{1},x_{2},x_{3}\right]=\left[n,f_{z},a_{p}\right]$$

#### 4.1.2. Optimization Objectives

The objective function reflects the optimization relationship between the optimization variables and each evaluation index. In the actual machining of CFRP components, a small cutting force and high processing quality and processing efficiency are required, so the cutting force, delamination damage, and material removal rate were taken as the optimization objectives.

The calculation formula of the cutting force in the three directions is shown in Equation (19). With the minimum cutting force as the optimization objective, the expression is as follows:

(27) $$f_{1}(X)=\min F_{T}=\min\sqrt{F_{x}^{2}+F_{y}^{2}+F_{z}^{2}}$$

CFRP materials in the milling process will produce many processing defects, such as delamination damage, roughness, etc. Delamination defects have the most significant impact on the stiffness, strength, and life of components. Therefore, the minimum delamination damage was taken as the optimization objective [34], and the delamination factor was calculated as shown in Equation (28).

(28) $$\begin{array}{cc} F_{d}=1.0706\;+ & 1.0575\;\times \;10^{-4}n\;+\;1.7875f_{z}\;+\;0.0995a_{p}\\ & +\;7.5\times 10^{-4}nf_{z}\;+\;5\;\times \;10^{-6}na_{p}\;+\;0.5f_{z}a_{p}\;-\;2.025\;\times \;10^{-8}n^{2}\\ & -\;31.875{f_{z}}^{2}\;-\;0.021{a_{p}}^{2} \end{array}$$

(29) $$f_{2}(X)=\min F_{d}$$

In the actual process, improving the processing efficiency is one of the goals of mechanical processing. On the premise of satisfying the processing quality, the processing efficiency should also be considered, so the material removal rate was used as the optimization goal.

(30) $$f_{3}(X)=\max Q=\max(nNf_{z}a_{p}d)$$

where Q denotes the material removal rate and d denotes the cutter’s diameter.

#### 4.1.3. Constraint Conditions

Considering the actual processing conditions and the content of previous literature [35], the restriction conditions were determined:

(31) $$st.\left\{\begin{array}{c} 1000\leq n\leq 5000\\ 0.5\leq a_{p}\leq 2\\ 0.01\leq f_{z}\leq 0.05 \end{array}\right.$$

### 4.2. Multi-Objective Optimization Based on the Genetic Algorithm

The MATLAB GA toolbox was mainly used in the optimization process. Additionally, in the optimization process, various factors were considered. Thus, the parameters were set as follows: the initial population size was set to 200, the optimal front-end individual coefficient was set to 0.3, the maximum genetic algebra was set to 300, the stop algebra was set to 300, the fitness function deviation was set to 1 × 10^−100^, and the gamultiobj function was called for calculation.

### 4.3. Discussion and Verification of the Results

**Figure 11:**
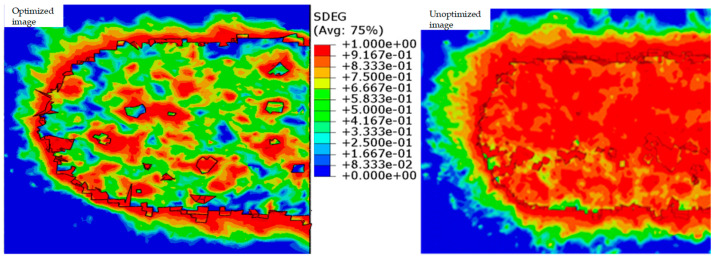
Comparison between after optimization and before optimization.

Table 10 shows the partial optimal solutions obtained. We can see that Scheme 2 is optimal, and it was verified by the simulation experiments. As shown in Figure 11, it can be clearly seen that the processing quality was improved.

As one can see from Table 7, if the minimum delamination factor is taken as the main objective, with Options 2, 11, and 12 for the machining parameters, the cutting conditions of high speed, a small feed rate, and a small cutting depth can be selected. If the maximum material removal rate is the main objective, with Options 1, 3, and 7 for the machining parameters, the cutting conditions of high speed, a large feed rate, and a large cutting depth can be selected. Therefore, the appropriate processing parameters should be selected according to the actual engineering requirements in actual processing.

**Figure 12:**
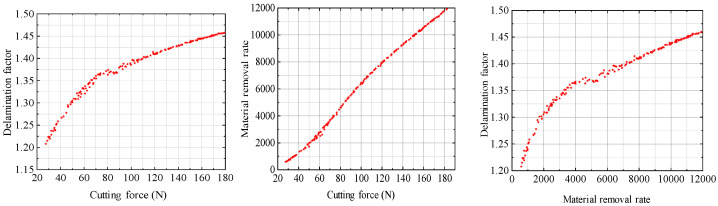
Relationship between cutting force and both the delamination factor and the material removal rate.

The relationship among the cutting force, the delamination factor, and the material removal rate are shown in Figure 12. As can be seen from the figure, when the cutting force is between 80 N and 100 N, the delamination factor is close to a fixed value, but below 80 N and above 100 N, the delamination factor increases approximately linearly with the cutting force, and after 100 N, the increase is slowed. Considering that the main factors affecting the cutting force are the feed rate and cutting depth per tooth, the spindle speed has little effect on the cutting force, while the delamination factor increases with increases in the feed rate and the cutting depth per tooth. Therefore, between 80 N and 100 N per tooth, the feed rate, cutting depth, spindle speed, and other influences are relatively balanced, resulting in a stable state of the delamination factor with the increase in cutting force. According to the analysis above, the cutting force is mainly affected by the spindle speed and cutting depth, and the cutting force and material removal rate obviously increase linearly with increases in the spindle speed and cutting depth. The material removal rate is affected by the spindle speed, cutting depth, feed rate per tooth, and other factors, showing a linear relationship, while the delamination factor increases with an increase in the feed rate per tooth and cutting depth, and decreases with an increase in spindle speed. Therefore, the relationship between the delamination factor and the material removal rate is more complicated.

## 5. Conclusions and Discussion

In this paper, a CFRP milling force prediction model considering the cutting action of an end milling cutter’s bottom edge was established, and the cutting force coefficient was calculated to predict the milling force by including the cutting force coefficient in the model. Then, based on the delamination damage and milling force prediction model, the optimization model was established by taking the machining parameters as the optimization variables, and the optimal machining processing parameters were obtained by the genetic algorithm. The conclusions are as follows.

(1) The relationships among spindle speed, feed rate per tooth, cutting depth and cutting force were obtained under different fiber directions, the cutting force coefficient was calibrated by the inverse method, and the relation between the cutting force coefficient and machining parameters was obtained. After processing the cutting force data obtained by experiments and predicted by model, the maximum relative error between the experimental and predicted cutting force was 14.5%, which indicates the correctness of the cutting force prediction model.
(2) Taking the unidirectional carbon fiber plate with a fiber direction angle of 45° as an example, and taking delamination damage, cutting force, and the material removal rate as the optimization objectives, the cutting parameters of CFRP were optimized to find the best processing schemes. In addition, optimization schemes were obtained when the optimization objectives were different. It can be concluded that when the minimum stratification factor is taken as the main objective, the cutting conditions of high speed, a small feed rate, and a small cutting depth can be selected. When the maximum material removal rate is taken as the main objective, the cutting conditions of a high speed, a large feed rate, and a large cutting depth can be selected. These studies will have practical guiding significance for the processing of CFRP.
(3) At the same time, the relationships among cutting force, the delamination factor, and the material removal rate were obtained; that is, with an increase in the cutting force, the material removal rate gradually increases, and the delamination factor gradually increases. In actual manufacturing, more conditions can be set for this multi-objective optimization model to facilitate enterprises in selecting the optimal parameters that suit their actual manufacturing capabilities.

In this study, the influence of spindle speed on the cutting force was ignored in the modeling, and the cutting force was regarded as a linear relationship with the feed of each tooth. In future work, multi-factor fitting should be considered. 

## Figures and Tables

**Table 1:** Mechanical properties and parameters of the unidirectional CFRP plate material.

Material Parameter	Value
Density—ρ (kg/m^3^)	1.81
Modulus of elasticity—E11 (GPa)	235
Modulus of elasticity—E22, E33 (GPa)	14
Poisson ratio—v12 = v13	0.2
Poisson ratio—v23	0.25
Tensile strength of Direction 1—XT (MPa)	4620
Tensile strength of Direction 2—YT (MPa)	1500
The compressive strength in Direction 1—XC (MPa)	3960
The compressive strength in Direction 2—YC (MPa)	3340
Modulus of shearing—G12, G13 (MPa)	28,000
Modulus of shearing—G23 (MPa)	55,000
Shear strength—S12, S13, S23 (MPa)	1500

**Table 2:** Simulation experiment’s parameters.

Fiber Direction Angle	Spindle Speed (rpm)	Feed Rate per Tooth (mm/z)	Axial Cutting Depth (mm)
0°, 45°, 90°, 135°	4000	0.03	1

**Table 3:** Experimental parameters of milling.

Fiber Direction Angle	Spindle Speed (rpm)	Feed Rate per Tooth (mm/z)	Axial Cutting Depth (mm)
0°/90°	3000/4000/5000	0.01/0.015/0.03/0.05	1/1.5

**Table 4:** Experimental parameters of the simulation.

Fiber Direction Angle	Spindle Speed (rpm)	Feed Rate per Tooth (mm/z)	Axial Cutting Depth (mm)
45°/135°	3000/4000/5000	0.01/0.015/0.03/0.05	1/1.5

**Table 5:** Fitting coefficients of cutting force under the condition of an axial cutting depth of 1 mm.

Machining Parameter	Spindle Speed, 4000 r/min			Cutting Depth, 1 mm		
	*x*		*y*		*z*	
Fiber direction angle	a	b	a	b	a	b
0°	37.9767	595.6194	42.5652	1095.5265	14.2917	65.17425
45°	41.7885	1457.9613	20.3633	1313.0761	16.4530	143.4955
90°	59.1815	1447.1342	27.1918	490.4374	15.0872	103.4310
135°	39.0930	1555.1226	33.5799	788.5097	12.9637	146.6194

**Table 6:** Cutting force fitting coefficients under the condition of an axial cutting depth of 1.5 mm.

Machining Parameter	Spindle Speed, 4000 r/min			Cutting Depth, 1.5 mm		
	*x*		*y*		*z*	
Fiber direction angle	a	b	a	b	a	b
0°	49.3791	1105.890	79.2082	985.24	14.9633	73.3032
45°	41.9662	2396.5987	45.1799	1278.8684	15.7889	190.2323
90°	73.9529	2233.5007	29.9825	815.2297	16.0592	122.4129
135°	70.8019	1912.3097	51.2523	878.3213	12.9908	179.5871

**Table 7:** Cutting force coefficients of different fiber direction angles.

Cutting Force Coefficient	Fiber Direction Angle			
	0°	45°	90°	135°
*K_rc_*	−1020.54	−1877.27	−1572.73	−714.37
*K_re_*	−27.83	−27.38	−42.58	−33.87
*K_tc_*	−220.57	−68.42	649.58	179.62
*K_te_*	37.43	19.81	18.52	26.59
*K_ac_*	12.76	73.38	29.80	51.76
*K_ae_*	6.07	6.74	6.45	5.41
*K_tb_*	4396.99	4604.97	−530.49	2029.62
*K_rb_*	1416.41	1397.71	418.66	−2802.49
*K_ab_*	127.99	130.89	171.31	211.12

**Table 8:** Experimental parameters.

Fiber Direction Angle	Spindle Speed	Axial Cutting Depth	Feed Rate per Tooth
0°	3000 r/min	2 mm	0.03 mm/min

**Table 9:** The relative error between the experimental and predicted milling force.

Milling Force (N)	x	y	z
Predicted value of milling force	119.381	121.619	26.71
Experimental value of milling force	106.95	118.323	23.32
Relative error	11.6%	2.9%	14.5%

**Table 10:** Partial Pareto optimal solution.

Serial Number	Spindle Speed	Feed Rate per Tooth	Axial Cutting Depth	Cutting Force	Delamination Factor	Material Removal Rate
	(r/min)	(mm/z)	(mm)	(N)		(mm^3^/min)
1	4969.76	0.05	2.00	182.19	1.459	11,927.4
2	4903.16	0.01	0.50	27.53	1.208	589.00
3	4961.63	0.049	1.99	177.26	1.457	11,606.7
4	4959.13	0.026	1.95	94.98	1.382	6006.9
5	4964.77	0.045	1.99	160.49	1.445	10,590.2
6	4966.39	0.04	1.99	142.35	1.430	9449.2
7	4969.33	0.047	1.99	171.47	1.453	11,252.6
8	4953.01	0.035	1.98	125.78	1.417	8313.9
9	4956.25	0.027	1.91	96.85	1.387	6134.7
10	4966.39	0.04	1.99	143.04	1.431	9494.1
11	4884.56	0.01	0.53	28.66	1.215	634.9
12	4887.84	0.0116	0.54	30.59	1.224	737.7

## Data Availability

The original contributions presented in the study are included in the article, further inquiries can be directed to the corresponding authors.

## References

[1] Liu Y.J., Li S.P., Li H., Qin X.D., Xing Y.Q., Liu H.B. (2020). The design and performance evaluation of assisted chip removal system in helical milling of CFRP/Ti stacks. Int. J. Adv. Manuf. Technol..

[2] Hou Y., Yao P., Zhang H.Y., Liu X., Liu H.L., Huang C.Z., Zhang Z.H. (2021). Chatter stability and surface quality in milling of unidirectional carbon fiber reinforced polymer. Compos. Struct..

[3] Yue C.X., Liu X.L., Liang S.Y. (2017). A model for predicting chatter stability considering contact characteristic between milling cutter and workpiece. Int. J. Adv. Manuf. Technol..

[4] Mao C.J., Liu K.Y., Cepero-Mejias F., Zhang C. (2024). Numerical investigation on milling performance and damage response of UD-CFRP laminates. Mech. Adv. Mater. Struct..

[5] Wang F.J., Li Y., Zhang B.Y., Deng J., Lin Y.Q., Yang L.L., Fu R. (2022). Theoretical model of instantaneous milling force for CFRP milling with a ball-end milling cutter: Considering spatial dimension and temporal dimension discontinuity effects. J. Manuf. Process..

[6] Wang S.J., Zhang T., Hu B.W., Miu G.Q., Sun Z.W., Sandy T. (2024). Analytical model for the prediction of milling forces: A review. Int. J. Adv. Manuf. Technol..

[7] He Y.L., Qing H.N., Zhang S.G., Wang D.Z., Zhu S.G. (2017). The cutting force and defect analysis in milling of carbon fiber-reinforced polymer (CFRP) composite. Int. J. Adv. Manuf. Technol..

[8] Wang H., Tao K., Jin T. (2021). Modeling and estimation of cutting forces in ball helical milling process. Int. J. Adv. Manuf. Technol..

[9] Shang S., Qin X.D., Li J.H., Li S.P., Li H., Huang T., Jin Y., Sun D. (2018). Modelling of cutting forces and researching calibration method in helical milling. Int. J. Adv. Manuf. Technol..

[10] Zhou L., Wang Y.L., An G.S., Zhu R.B., Li G.Q., Ma R. (2024). Multi-objective grey correlation analysis based on CFRP Helical Milling simulation model. Int. J. Adv. Manuf. Technol..

[11] Li Z.Q., Liu Q., Ming X.Z., Wang X., Dong Y.F. (2014). Cutting force prediction and analytical solution of regenerative chatter stability for helical milling operation. Int. J. Adv. Manuf. Technol..

[12] Chen R., Li S.J., Li P.N., Liu X.P., Qiu X.Y., Ko T.J., Jiang Y. (2020). Effect of fiber orientation angles on the material removal behavior of CFRP during cutting process by multi-scale characterization. Int. J. Adv. Manuf. Technol..

[13] Ning H.F., Zheng H.L., Wang G.X. (2022). Establishment of Analytical Model for CFRP Cutting Force Considering the Radius of the Edge Circle. Materials.

[14] Wang H., Pei Z.J., Cong W.L. (2020). A feeding-directional cutting force model for end surface grinding of CFRP composites using rotary ultrasonic machining with elliptical ultrasonic vibration. Int. J. Mach. Tools Manuf..

[15] Kim D.G., Yang S.H. (2023). Efficient Analysis of CFRP Cutting Force and Chip Formation Based on Cutting Force Models Under Various Cutting Conditions. Int. J. Precis. Eng. Manuf..

[16] Zhang S., Jiao F., Wang X., Niu Y. (2021). Modeling of cutting forces in helical milling of unidirectional CFRP considering carbon fiber fracture. J. Manuf. Process..

[17] Sheikh-Ahmad J., He Y.L., Qin L. (2019). Cutting force prediction in milling CFRPs with complex cutter geometries. J. Manuf. Process..

[18] Ning H.F., Zheng H.L., Yuan X.M. (2021). Establishment of instantaneous milling force prediction model for multi-directional CFRP laminate. Adv. Mech. Eng..

[19] Wan M., Zhang W.H., Yang Y. (2011). Phase width analysis of cutting forces considering bottom edge cutting and cutter runout calibration in flat end milling of titanium alloy. J. Mater. Process. Technol..

[20] Ozsoy N., Eksi S., Ozsoy M. (2023). Cutting parameters optimization of hybrid fiber composite during drilling. Mater. Test..

[21] Mahdi A., Makhfi S., Habak M., Turki Y., Bouaziz Z. (2023). Analysis and optimization of machining parameters in drilling woven carbon fiber reinforced polymer CFRP. Mater. Today Commun..

[22] Barik T., Parida S., Pal K. (2024). Optimizing Process Parameters in Drilling of CFRP Laminates: A Combined MOORA–TOPSIS–VIKOR Approach. Fibers Polym..

[23] Li J., Yang X.Y., Ren C.Z., Chen G., Wang Y. (2015). Multiobjective optimization of cutting parameters in Ti-6Al-4V milling process using nondominated sorting genetic algorithm-II. Int. J. Adv. Manuf. Technol..

[24] Shen Y.F., Yang T., Liu C., Liu S.N., Du Y. (2021). Cutting force modeling in orthogonal cutting of UD-CFRP considering the variable thickness of uncut material. Int. J. Adv. Manuf. Technol..

[25] Wan M., Du Y.X., Zhang W.H., Yang Y. (2021). Cutting force modeling in helical milling process of unidirectional CFRP. Acta Aeronaut. Astronaut. Sin..

[26] Budak E., Altintas Y., Armarego E.J.A. (1996). Prediction of Milling Force Coefficients from Orthogonal Cutting Data. ASME. J. Manuf. Sci. Eng..

[27] Ali M.N., Khalil H., El-Hofy H. (2024). Analytical modeling of cutting force in vibration-assisted helical milling of Al 7075 alloy. J. Manuf. Process..

[28] Fan J., Guan Z.W., Cantwell W.J. (2014). Numerical modelling of perforation failure in fibre metal laminates subjected to low velocity impact loading. Compos. Struct..

[29] Cepero-Mejías F., Phadnis V.A., Kerrigan K., Curiel-Sosa J.L. (2021). A finite element assessment of chip formation mechanisms in the machining of CFRP laminates with different fibre orientations. Compos. Struct..

[30] Lu X., Ridha M., Chen B.Y., Tan V.B.C., Tay T.E. (2019). On cohesive element parameters and delamination modelling. Eng. Fract. Mech..

[31] Krishnaraj V., Prabukarthi A., Ramanathan A., Elanghovan N., Senthil Kumar M., Zitoune R., Davim J.P. (2012). Optimization of machining parameters at high speed drilling of carbon fiber reinforced plastic (CFRP) laminates. Compos. Part B Eng..

[32] Liu L.L., Yao P., Zhang H.Y., Chu D.K., Qu S.S., Huang C.C. (2024). Laser trimming and performance analysis of mill-grinding tools for carbon fiber–reinforced plastics. Int. J. Adv. Manuf. Technol..

[33] Su C.J., Cheng X., Yan X.H., Zheng G.M., Yang L., Mu Z.G. (2022). Helical milling for making holes on carbon fiber-reinforced polymer. Int. J. Adv. Manuf. Technol..

[34] Zhang H.Y., Zhu P., Liu Z., Qi S.J., Zhu Y.D. (2020). Research on prediction method of mechanical properties of open-hole laminated plain woven CFRP composites considering drilling-induced delamination damage. Mech. Adv. Mater. Struct..

[35] Liu J., Chen G., Ji C.H., Qin X.D., Li H., Ren C.Z. (2014). An investigation of workpiece temperature variation of helical milling for carbon fiber reinforced plastics (CFRP). Int. J. Mach. Tools Manuf..

